# Changes in Biomarkers of Inflammation and Oxidative Status in Dogs Subjected to Celiotomy or Video-Assisted Ovariohysterectomy

**DOI:** 10.3390/vetsci11110583

**Published:** 2024-11-19

**Authors:** Fabíola Dalmolin, Camila Peres Rubio, Carla Sordi Furlanetto, Rafael Steffens, Najla Ibrahim Isa Abdel Hadi, Adriellen de Lima da Silva, Paloma Tomazi, Bernardo Nascimento Antunes, Fabiana Elias, Elizabeth Moreira dos Santos Schmidt, Maurício Veloso Brun

**Affiliations:** 1 Programa de Pós-Graduação em Saúde, Bem-estar e Produção Animal Sustentável na Fronteira Sul (PPG-SBPAS), Universidade Federal da Fronteira Sul (UFFS), Realeza 85770-000, Brazil; fabiola.dalmolin@uffs.edu.br (F.D.); carla.sordi@hotmail.com (C.S.F.); rafaelsteffens@msn.com (R.S.); najlahadi@hotmail.com (N.I.I.A.H.); palomatomazi.medvet@gmail.com (P.T.); 2 Department of Animal Surgery and Medicine, University of Murcia, 30100 Murcia, Spain; 3 Curso de Medicina Veterinária, Universidade Federal da Fronteira Sul (UFFS), Realeza 85770-000, Brazil; adriellenlima97@gmail.com (A.d.L.d.S.); fabiana.elias@uffs.edu.br (F.E.); 4 Programa de Pós-Graduação em Medicina Veterinária (PPGMV), Universidade Federal de Santa Maria (UFSM), Santa Maria 97105-900, Brazil; bernardonascimentoantunes@gmail.com (B.N.A.); mauriciovelosobrun@hotmail.com (M.V.B.); 5 School of Veterinary Medicine and Animal Science, São Paulo State University (FMVZ-UNESP), Campus Botucatu, Botucatu 18618-681, Brazil; elizabeth.schmidt@unesp.br

**Keywords:** homeostasis, inflammation, laparoscopy, neutering, oxidative stress

## Abstract

We evaluated the surgical stress response of dogs undergoing three ovariohysterectomy (OVH) techniques. Twenty-nine healthy females were allocated into groups: celiotomy and ligature (CelioSut), celiotomy and bipolar energy (CelioBip), and a video-assisted technique using two portals and bipolar energy (VidBip). Clinical evaluation was performed, and the following blood analyses were determined: acute phase proteins (C-reactive protein and haptoglobin), white blood cell counting (WBC), and biomarkers of oxidative status. The VidBip required more time despite a smaller incision, lower heart rate, and earlier feeding and urination. All groups had high white blood cells counts; the C-reactive protein (CRP) levels peaked at 6 and 12 h in all groups and was higher in the CelioBip and VidBip groups compared to CelioSut; haptoglobin concentrations peaked at 48 h in all groups and increased in dogs of the CelioSut group. Trolox equivalent antioxidant capacity, ferric reducing ability of plasma, cupric reducing antioxidant capacity, and advanced oxidation protein were not significantly different among the groups or time points. Total thiol concentrations were lower in CelioBip and CelioSut groups. All surgical techniques induced an inflammatory and oxidative stress response, but the video-assisted technique produced early clinical recovery. The bipolar device produces fewer disturbances than suspensory ligament rupture and ligature.

## Simple Summary

There are contrasts in the recovery and response to surgical stress in canine after open and video surgery. The main difference includes incision size, intra- and postoperative pain, recovery time regarding eating, urination, defecation, and movement, as well as changes in acute phase proteins, oxidative metabolism, and leukocytosis, among others, with advantages to laparoscopy. The aim of this study was to clarify the effect of the access and hemostatic techniques regarding inflammation and oxidative stress. There is some evidence that video surgery, due to the carbonic gas, provokes higher oxidative stress regardless of lower inflammation compared to open surgery. This study was carried out including one of the most used techniques in video surgery, the video-assisted two portals technique, a conventional technique involving manual rupture of the ovarian suspensory ligament and ligation with surgical threads and an open access without ligament rupture and bipolar coagulation. This study demonstrates similar oxidative stress between techniques and advantages to the video-assisted technique, even when using a pneumoperitoneum.

## 1. Introduction

The ovariohysterectomy (OVH) is one of the most common surgeries performed in dogs [1,2]. It can be accessed in several ways, such as open midline abdominal incision and laparoscopy [1,3,4], and can leverage various hemostatic methods such as a suture ligature, vascular clips, and bipolar energy, among others [1,3].

Previous studies in dogs have evaluated the response to surgical stress to understand the effects of different techniques and to identify, manage, or prevent complications [4,5,6]. In general, patients experience trauma and anesthesia-induced immune activation in the postoperative period, which exacerbates the inflammatory response [4]. Inflammation is a fundamental defense mechanism against infection and the initiator of important tissue repair. In contrast, prolonged and uncontrolled inflammation can have negative effects such as excessive pain, immunosuppression, organ dysfunction, and death [7]. The response to surgery consists of hormonal, metabolic, and inflammatory responses that occur after surgery and allow the body to adapt to trauma and repair damaged tissues [8].

During the acute phase response, there is an increase in the synthesis and release of acute phase proteins, driven by proinflammatory cytokines produced primarily by the liver, such as C-reactive protein (CRP) and haptoglobin (Hp) [9]. In addition to inflammation, oxidative stress due to surgical procedures has also been shown in dogs [5,6]. Oxidative stress is an imbalance between oxidants and antioxidants in favor of oxidants, leading to disruption of redox signaling and control and/or molecular damage [10]. A change in the oxidative balance is recognized by cell damage and the cause of several diseases. In major surgeries, it occurs acutely mainly during ischemia followed by reperfusion, as shown after laparoscopy [11]. The choice of intraoperative approaches, techniques, and materials often depends on efforts to reduce trauma [7] and shorten operative time [2]. Minimally invasive procedures, such as video-assisted procedures, had advantages over open surgery, resulting in less tissue injury and reduced release of inflammatory mediators, reduced pain, and thus early recovery [12], because it reduces the inflammatory response [3,5]. The hemostatic harmonic scalpel is an effective and efficient tool for hemostasis, although its cost is high. Bipolar electrocoagulation has been used instead because it is considered safe, less expensive [6], and promotes a shorter operative time and rare complications compared to conventional suspensory ligament and ligament rupture [2]. A bipolar device has been described in open [2] and video-assisted canine OVH [3,6].

The effect of traditional and bipolar hemostatic methods and the celiotomy and endoscopic access on inflammation and the oxidative response after canine OVH is unknown. Thus, the objectives of this study were the following: (i) to evaluate the dog’s response to surgical stress after OVH with mid-abdominal celiotomy and hemostatic ligature (CelioLig); ventral median celiotomy and bipolar coagulation (CelioBip); and video-assisted dual port and bipolar coagulation (VidBip); and (ii) to investigate the effect of different approaches (celiotomy and endoscopy) and hemostatic techniques (ligature and bipolar coagulation) on the surgical stress response of OVH in dogs by evaluating blood analytes such as acute phase protein concentrations (CRP and Hp), WBC, and the oxidative stress biomarkers (total antioxidant capacity (TAC), Trolox equivalent antioxidant capacity (TEAC), copper-reducing antioxidant capacity (CUPRAC), ferric reducing ability of plasma (FRAP), serum total thiol, and the advanced oxidation protein products biomarker (AOPP).

## 2. Materials and Methods

### 2.1. Animals

All the animal handling and procedures were approved by the Faculty’s Animal Experimentation Ethics Committee of the Universidade Federal da Fronteira Sul, Brazil (protocol number 23205.003951-2018-77 CEUA). The study population consisted of twenty-nine healthy female dogs of different breeds. The animals included were considered healthy based on physical and laboratory examinations (total white blood count (WBC), serum activity of alkaline phosphatase, alanine aminotransferase, serum concentrations of creatinine, and total plasma protein measurements). Those with a history of previous illness, skin abnormalities, ectoparasites, endoparasites, or chronic conditions (e.g., dermatological, respiratory, allergic, gastrointestinal) were excluded. All selected animals were dewormed, vaccinated, and limited to 4 years of age or younger to avoid issues related to degenerative or age-associated diseases. Abdominal ultrasound (Sonosite M Turbo, Bothell, United State), electrocardiogram (InCardio X InPulse, Florianópolis, Brazil), absence of significant macroscopic changes in the uterus, ovaries, and uterine tubes after surgery were also considered healthy. Patients were randomly divided into three groups according to the surgical procedure: i. CelioLig (*n* = 9, 2.05 ± 0.80 years; 15.40 ± 3.35 kg); ii. CelioBip (*n* = 10, 2.44 ± 1.50 years; 14.16 ± 3.73 kg); and iii. VidBip (*n* = 10, 1.80 ± 0.63 years; 13.26 ± 3.33 kg).

### 2.2. Surgical Preparation and Anesthesia

Forty-eight hours before surgery, patients were placed in boxes in the same location where postoperative assessments were performed. Animals were given water and food ad libitum and had access to an external restricted area for defecation and urination. Patients were exposed to researchers and other laboratory animals only 96 h after surgery. The same team performed all procedures, avoiding pattern changes. After a 12 h food and 8 h water fast, the dogs received acepromazine (0.05 mg/kg i.m.), (Acepran 0.2%, Univet, São Paulo, Brazil), and 15 min later, a broad trichotomy of the abdomen and venous access with Ringer’s Lactate solution (5 mL/kg/h i.v.), (Equiplex, Aparecida do Goiás, Brazil) were performed. The anesthesia was induced via propofol (6 mg/kg) administered intravenously (IV), (Fresofol, Frenesius-Kabi, São Paulo, Brazil) followed by tracheal intubation and 100% oxygen vaporized isoflurane in a calibrated vaporizer. Fentanyl (2.5 µg/kg IV), (Janssen-Cilag Farmacêutica LTDA, São Paulo, Brazil) analgesic was used before the right ovary manipulation. Morphine was predicted if an elevation of 30% or more of the initial cardiac rate or systolic blood pressure happened. During anesthesia, a 16 G catheter was introduced into the jugular vein, fixed via a suture and instantaneous adhesive and protected by a sterile bandage to obtain blood samples at the postoperative time points.

### 2.3. Surgical Procedures

All procedures were started with patients in dorsal recumbency. The CelioSut group underwent a retro-umbilical celiotomy involving one-third of the distance between the umbilicus and the pubis. After manual rupture of the ovarian suspensory ligament, polyglactin 910 (Shalon, Goiás, Brazil) double ligatures (circular and fixed) were applied to the ovarian pedicles and uterine body using a modified three-clamp technique [12]. Celiorrhaphy was performed with the same thread and cross mattress pattern. Subcutaneous reduction was performed with continuous suture and the same material, and dermorraphy was performed with an isolated horizontal mattress template and nylon suture. CelioBip was performed via celiotomy. Additionally, 5 mm laparoscopic bipolar forceps (Gyrus ACMI^®^, Southborough, MA, USA) were used for hemostasis of the ovary and uterine body. No ovarian suspensory ligament rupture was performed in this group. Abdominal sutures were performed as described above.

For VidBip, we used two ports (11 mm; length 10.5 cm), one through the umbilicus and the other in the pre-pubic region. The abdomen was inflated with CO_2_ (1/min) to 10 mmHg and a 10 mm 0° rigid endoscope was used. Patients were placed in the lateral recumbent position, and the ovary was temporarily fixed to the abdominal wall with a transparietal suture using a traumatic curved needle (2 cm) and 2-0 nylon. Hemostasis was performed via bipolar coagulation as in CelioBip. Vascular hemostasis of the uterine body was performed extracorporeally. Residual gas was removed, and abdominal sutures were applied as described above [3]. The dimensions of the sutures were adjusted according to the size of the patient. The size of the incisions was measured with a pachymeter; in the VidBip group, the size of two incisions was recorded.

### 2.4. Postoperative and Blood Sampling

Immediately after surgery, patients received tramadol hydrochloride (5 mg/kg subcutaneously, every 8 h/48 h) (Medley Indústria Farmacêutica Ltd.a, Campinas, São Paulo), chlorhexidine for wounds (every 24 h), and protective clothing. The patients were evaluated at 2, 6, 12, 24, and 48 h after surgery with a pain scale [13] and analgesic rescue was predicted with morphine (0.3 mg/kg intramuscularly) if the patients reached a score of six or more points.

Rectal temperature, respiratory rate (RR), heart rate (HR), and blood samples were taken immediately before surgery and at 2, 6, 12, 24, 48 h and seven days later. For WBC count, 0.5 mL of blood was collected via jugular venipuncture and placed in EDTA tubes (Labor 0.5 mL) in the same time points and processed immediately. Another aliquot (5 mL) was obtained, placed in a serum separator tube, and centrifuged at 3000× *g* for 10 min. Each serum sample was aliquoted (400 µL/Eppendorf type tube) and stored at −80 °C until analysis. Frozen aliquots (−80 °C) were sent to INTERLAB—University of Murcia (UMU) for analysis of biomarkers of inflammation and oxidative stress.

### 2.5. Laboratory Analysis

White blood cells were automatically counted (Bio–2900 Vet—Bioeasy Diagnostical, São Paulo, Brazil). Differential leukocyte counts were performed via slide preparation stained with the Diff Quick method and an optical microscope reading which included segmented neutrophils, lymphocytes, monocytes, eosinophils, and basophils [14].

Serum C-reactive protein (CRP) concentration was determined via immunoturbidimetric analysis (CRP OSR6147, Olympus Life and Material Science, Lismeehan, Ireland). Serum haptoglobin (Hp) concentration was measured using a commercial kit (Tridelta Development, Maynooth, Ireland). The methods were previously validated for use in dogs [14,15]. Biomarkers of oxidative status previously validated in canine serum samples were determined: total antioxidant capacity (TAC) [16,17], Trolox equivalent antioxidant capacity (TEAC) [16,18], copper-reducing antioxidant capacity (CUPRAC) [18,19], ferric reducing ability of plasma (FRAP) [20,21], and serum total thiol [22,23]. Products of oxidation of proteins were evaluated via the advanced oxidation protein products biomarker (AOPP) [24]. The analyses were performed using an automated biochemistry analyzer (Olympus AU400, Olympus Diagnostica GmbH, Hamburg, Germany).

### 2.6. Statistical Analysis

Data were assessed for their distribution pattern with the Shapiro–Wilk test and for homogeneity of variances with the Levene test. Comparisons between groups were made using two-way ANOVA and Tukey’s multiple comparison test. Comparison between time-points was performed using two-way ANOVA and Dunnett’s multiple comparison test. Normally distributed data were presented as means ± standard deviation. Nonparametric data were presented as median and interquartile range. Differences were considered significant at 5% (*p* < 0.05).

## 3. Results

### 3.1. Surgery Data

All animals had no surgical complications, and all recovered after eight days. During the CelioSut surgery, a spontaneous pressure opening was observed in the uterine body (*n* = 1). Sharp paramedian incision (*n* = 2), bipolar device damage to the peritoneum (estimated as small as 0.3 cm) (*n* = 1), and difficulty with ovarian exposure (*n* = 2) were noted during CelioBip. In addition, events included a VidBip-shaped paramedian portal (*n* = 3), portal displacement (*n* = 3), transparietal suture difficulty (*n* = 5), superficial splenic injury (*n* = 2), bipolar peritoneal injury (*n* = 4) (estimate as small as 0.2 cm), and difficulties with exteriorization of the uterus and the ovaries (*n* = 2); these events occurred mainly in two obese and large dogs, which increased the difficulty of the procedure and the surgical time. In the endoscopic surgery, a longer operative time (*p* < 0.01) and smaller incision (*p* < 0.01) were observed.

### 3.2. Animal Recovery and Clinical Examination

Analgesic rescue was not necessary for any animal during the evaluation time. Earlier time to first ingestion of solids (*p* < 0.01) and urination (*p* = 0.03) were observed in the VidBip group compared to others (Table 1). The first urination was earlier in the CelioBip group than in the CelioSut group (*p* = 0.03). No difference was found between groups in time to first bowel movement (*p* = 0.28) (Table 1). Regarding clinical parameters, rectal temperature was significantly different between groups between 6 h (*p* = 0.04) and 12 h (*p* = 0.04); hypothermia was observed in CelioSut and CelioBip and was more pronounced in the latter. Over time, CelioSut (37.4 and 38 °C, *p* < 0.01) and CelioBip animals (37.7 and 37.9 °C, *p* = 0.03) showed hypothermia (range 38–39 °C) at two and six hours. VidBip showed a normal temperature (~38 °C, *p* = 0.01). Regarding RR, VidBip animals had a lower effect at 24 h compared to other groups (*p* = 0.01). No difference was observed between CelioSut and CelioBip (*p* > 0.05). Regarding HR, a statistically significant difference was observed between groups at 6 h (*p* = 0.01), 12 h (*p* < 0.01), 24 h (*p* < 0.01), and 48 h (*p* = 0.03); VidBip animals showed lower values, and no differences were observed between the other groups. Over time, only VidBip animals showed a difference; after baseline, HR decreased at 6, 12, and 24 h and increased at 48 h (*p* < 0.01) (Table 2).

### 3.3. Laboratorial Analysis

**Figure 1:**
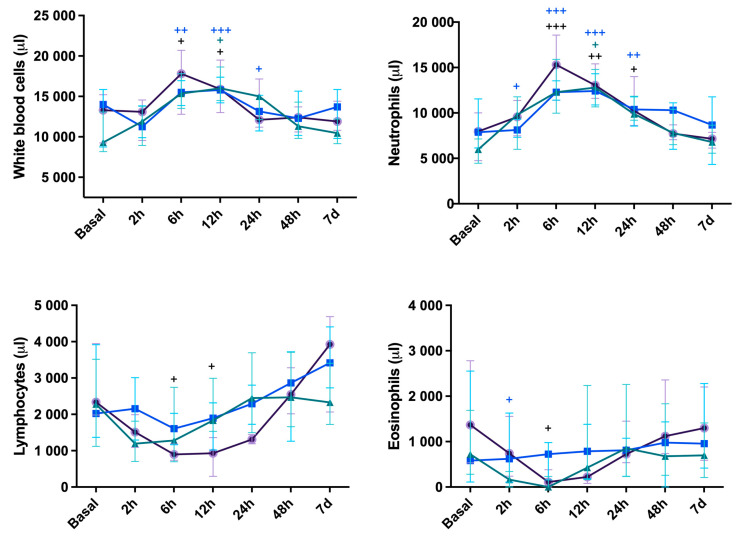
Total leukocytes, neutrophils, and lymphocytes median score and eosinophils median score in canines submitted to ovariohysterectomy via celiotomy and ligature (CelioSut; purple lines), celiotomy and bipolar coagulation (CelioBip; green lines), or the video-assisted technique with two ports and bipolar coagulation (VidBip; blue lines) along the evaluation time. Plus (+) means difference between the time-point and the basal time (+, *p* < 0.05; ++, *p* < 0.01; +++, *p* < 0.001).

White blood cell count is shown in Figure 1. Over time, WBC counts peaked at 6 h and 12 h for CelioSut (*p* < 0.05) and returned to the base line at 24 h; the CelioSut WBC counts increased at 6 h (*p* < 0.01), peaked at 12 h (*p* < 0.001), and decreased at 24 h (*p* < 0.05), returning to the base line at 48 h; the VidBip group had no alteration. The segmented neutrophil counts in CelioSut peaked at 6 h (*p* < 0.001), decreased at 12 h (*p* < 0.01) and at 24 h (*p* < 0.05), and returned to basal levels at 48 h; the CelioBip group presented a difference at 2 h (*p* < 0.05), peaked at 6 h (*p* < 0.001) and 12 h (*p* < 0.001), reduced at 24 h (*p* < 0.01), and returned to the basal level at 48 h. The VidBip group increased just at 12 h (*p* < 0.05) and returned to normal at 24 h. Lymphopenia and eosinopenia was observed just at 6 h in CelioSut animals (*p* < 0.05). The lymphocytes for the CelioSut and CelioBip groups decreased at 2 h, 6 h, and 12 h, followed by increases at 48 h and seven days (*p* < 0.01; *p* < 0.01). No difference was observed for VidBip (*p* = 0.08). There were no differences in eosinophils between groups (*p* > 0.06). Over time, CelioSut animals had eosinopenia at 6 and 12 h (*p* < 0.01), followed by an increase at 24 and 48 h and a return to baseline at 48 h. There were no differences between groups or times for monocytes (*p* > 0.10).

**Figure 2:**
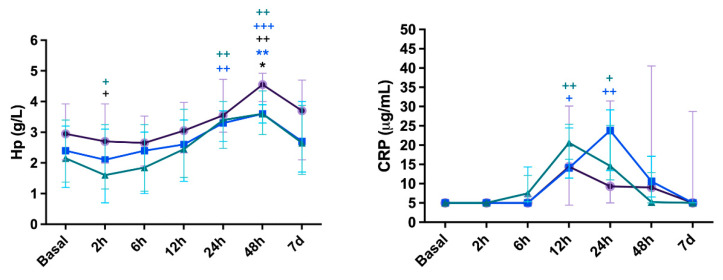
Haptoglobin (Hp) and C-reactive protein (CRP) concentrations in canines submitted to ovariohysterectomy via celiotomy and ligature (CelioSut; purple lines), celiotomy and bipolar coagulation (CelioBip; green lines), or the video-assisted technique with two ports and bipolar coagulation (VidBip; blue lines) along the evaluation time. Black asterisk (*) indicates difference between CelioSut and VidBip (*p* < 0.05). Blue asterisks (**), indicate differences between CelioSut and CelioBip (*p* ≤ 0,01). Plus (+) means a difference between the time-point and basal time (+, *p* < 0.05; ++, *p* < 0.01; +++, *p* < 0.001).

The results of inflammatory biomarkers are presented in Figure 2. Over time, CelioSut animals increased haptoglobin (Hp) concentration at 2 h and peaked at 48 h (*p* < 0.05; *p* < 0.01, respectively). The CelioBip increased at 2 h, peaked at 24 h and 48 h (*p* < 0.05, *p* < 0.01, *p* < 0.001), and returned to basal levels at 7 days. VidBip increased and peaked at 24 h and 48 h (*p* < 0.01). The differences between groups were at 48 h, with the CelioSut group being higher than CelioBip (*p* < 0.01) and with CelioSut higher than VidBip (*p* < 0.5).

In relation to the CRP levels, the CelioBip group showed increased levels at 12 h and 24 h after surgery (*p* < 0.01). Regarding the VidBip group, differences were identified at 12 h and 24 h (*p* < 0.05), and then CRP returned to baseline values. No significant differences were observed between groups for CRP (*p* > 0.05).

**Figure 3:**
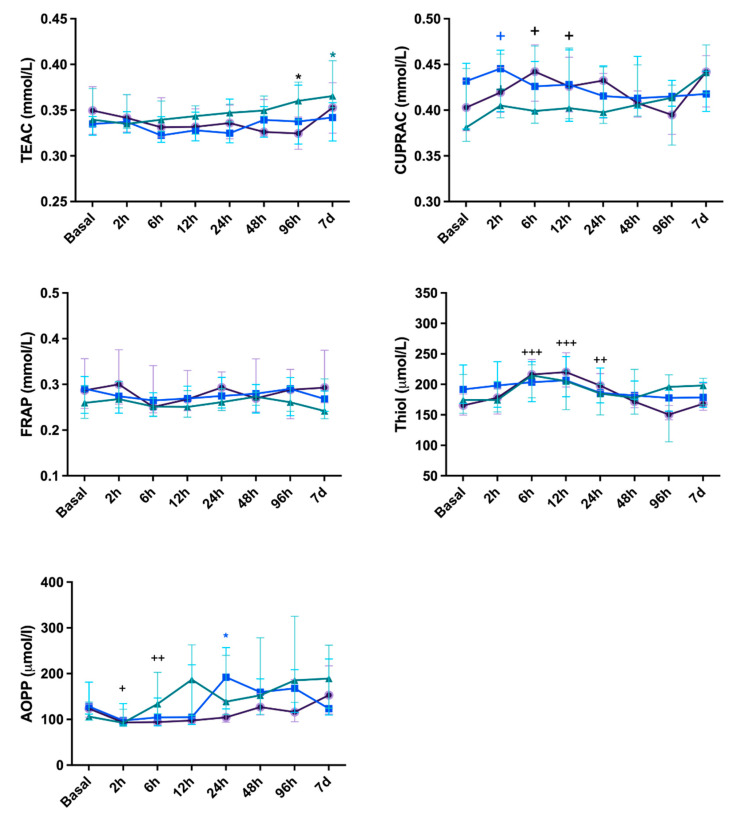
Trolox equivalent antioxidant capacity (TEAC), cupric reducing antioxidant capacity (CUPRAC), ferric reducing ability of plasma (FRAP), and thiol and the advanced oxidation protein products biomarker (AOPP) concentration in canine submitted to ovariohysterectomy via celiotomy and ligature (CelioSut; purple lines), celiotomy and bipolar coagulation (CelioBip; green lines), or the video-assisted technique with two ports and bipolar coagulation (VidBip; blue lines) immediately before surgery until seven days after. Black asterisks mean difference between CelioSut and VidBip; green asterisks mean difference between CelioBip and VidBip; blue asterisks mean difference between CelioSut and CelioBip (*p* < 0.05). Plus (+) means difference between the time-point and basal time (+, *p* < 0.05; ++, *p* < 0.01; +++, *p* < 0.001).

Considering the oxidative stress biomarkers (Figure 3), the TEAC increased at 96 h in CeliSut (*p* < 0.05) and at 7 days in CelioBip (*p* < 0.05); while in the VidBip group, TEAC presented no alteration. Regarding CUPRAC, in the CelioBip group the level was higher at 2 h (*p* < 0.05) and in the CelioSut at 6 h and 12 h (*p* < 0.05); the VidBip group showed no alteration in TEAC. FRAP showed no differences between the different time-points of the surgical procedures. The thiol was higher in the CelioSut at 2 h, 12 h, and 24 h (*p* < 0.001, *p* < 0.001 and *p* < 0.01, respectively) but showed no changes in the CelioBip and VidBip groups. The AOPP was higher at 6 h and 12 h in the CelioSut (*p* < 0.05) group, and at 24 h in VidBip (*p* < 0.05); however, in the CelioBip group, there were no differences.

## 4. Discussion

This study proposed to compare surgical stress after three OVH techniques, with different access and hemostatic methods, considering the importance of rapid recovery and fewer postoperative complications after a routine surgery in canines. Different OVH techniques have advantages and disadvantages, and they are described for female dogs [1,2,12]. In previous studies, the incidence of minor intraoperative complications after conventional canine OVH ranged from 7.5% to 19%. They were mainly associated with incision site inflammation and gastrointestinal upset [12]. Major complications included granulomas in remnants of the uterine or ovarian pedicle (28%). The complications which required intervention or treatment included inflammation or infection (8 of 142 dogs; 5.6%) and bleeding (4 of 142 dogs; 2.8%) [12]. Although studies with a large number of dogs are not available in the literature, the complication rate after laparoscopy of OVH was approximately 2% [4].

In our results, all techniques had small intercurrences during the procedures, which were more frequent in the VidBip group, probably because the surgeon had more practice in celiotomy than in celioscopy [6]. For the same reason, a longer operating time was required for the VidBip technique; probably due to the presence of two obese dogs (86 min and 74 min), a portal introduced to the vesical median ligament (86 min), and ovarian vascular coagulation difficulties in one patient (76 min) as previously reported [3]. Additionally, just a small needle size (2 cm) was available for the transparietal suture [3] and the lack of a specific surgical table for endoscopic surgery, which allows aseptic care and faster repositioning [6], may have lengthened the video-assisted procedure in this study.

When comparing the CelioSut and CelioBip groups, the advantage of the second one is that bipolar energy avoids foreign bodies entering the abdomen compared to suture material [2] and suture complications [1]. Bipolar electrosurgical devices can safely block vessels up to seven millimeters in diameter and uterine bodies below nine millimeters [12]. In this study the use of bipolar coagulation and ligature was equally difficult because the same instrument (video surgical bipolar) was required. Although, it has been associated with reduced surgical time when using a hand instrument [1,2]. Thus, the careful use of the laparoscopic device in open surgery was necessary due to the length of the instrument; the same device was used for the open and the video surgery to standardize the coagulation trauma. Ovarian exposure was difficult in CelioBip animals, and the surgical procedure took longer to perform, mainly in younger dogs, presumably due to the no rupture of the ovarian suspensory ligament [6].

Lower HR and RR were observed after laparoscopy compared to open procedures, which may be related to lower stress-induced cortisol and adrenaline release [25]. Mild hypothermia was observed after celiotomy, in contrast to normothermia after endoscopy. In humans, perioperative hypothermia is associated with higher intraoperative hemorrhage, adverse events such as surgical infection, longer postoperative chills, postanesthetic recovery, and hospitalization, although mortality is significantly increased [26]. Although hypothermia was more severe in this study when the bipolar device was used, no complications were observed probably due to the healthy conditions of the dogs [26]. In addition, the duration of anesthesia was associated with hypothermia [26], which was not observed in this study after laparoscopy, probably because the abdominal organs were not exposed, the low blood loss [3], and the position of the animals in lateral recumbency which minimizes body temperature changes [26]. The first solid intake and urination occurred earlier after video-assisted OVH than celiotomies, as previously described [4,5,6]. The video-assisted OVH is associated with less manipulation and injury, resulting in less cortisol and catecholamine release [26] and less pain. Thus, it promotes early recovery compared to open procedures [3,5].

In this study, leukocytosis was observed at 6, 12, and 24 h after surgery, being one of the changes observed for the acute inflammatory response in dogs [27]. The WBC peaked at 6 h to 24 h and returned to baseline values in all groups seven days after surgery, and both OVH techniques induced prolonged neutrophilia. The number of neutrophils increased and decreased similarly to WBC values due to a systemic inflammatory response [6,25,27]. In this study, lymphopenia and eosinopenia were observed just for the CelioSut group and were thus associated with the suspensory ligament rupture and ligature application. This result reinforces previous data, that the stress hemogram is present in canines subjected to open OVH [5].

The acute phase response is considered a part of the innate host defense system. The systemic inflammatory response is induced by the release of pro-inflammatory cytokines (interleukin-1, interleukin-6, and alpha tumor necrosis factor), which stimulate the synthesis and release of CRP and Hp by the liver [9]. In this study, haptoglobin concentrations increased 48 h after CelioSut compared to other groups, which could be related to suspensory ligament trauma [6]. The CRP concentrations which peaked at 12 h in CelioSut and at 24 h in VidBip, being higher for the CelioSut group, could be associated with surgical trauma [4], suggesting an initial although reduced inflammatory response for video surgery. Our results are similar to those previously described, where lower and higher CRP concentrations were associated with laparoscopy and open surgery, respectively [5,25]. Significant differences in CRP concentrations were observed over time when bipolar coagulation was applied via celiotomy and the video-assisted technique. A peak of CRP concentrations was observed at 6 and 12 h after surgery, suggesting that this protein was useful for monitoring patients after bipolar coagulation.

An imbalance between oxidants and antioxidants has been associated in humans with postoperative complications such as myocardial injury, sepsis, pulmonary edema, renal and hepatic failure, and increased mortality [28]. In this study, oxidative status was assessed using different integrated markers according to previous recommendations [21,28]. The antioxidant response can be monitored via the TAC assay, which is used to assess antioxidant levels in biological samples and to assess the antioxidant response against free radicals [17]. More specifically, it has been described that the thiol group is mainly responsible for the antioxidant effect of plasma proteins [22]. To the author’s knowledge, the combination of oxidative and antioxidant biomarkers used here has not been evaluated in dogs or humans in order to compare open and video-assisted surgery with the bipolar coagulation and ligature method.

No significant differences were found for FRAP in this study. The CUPRAC increased at 2 h in the CelioBip group and at 6 h and 12 h in the CelioSut group and the thiol concentrations increased at 6 h, 12 h, and 24 h for the CelioSut group, similar to the changes in WBC and neutrophils in the animals that had open surgery, confirming that oxidative stress and inflammation are part of the surgical stress response [7,10]. The AOPP increased at 6 h and 12 h for the CelioSut group, and it could be associated with the higher trauma of abdominal access and disruption of the suspensory ligament, as observed with the thiol, TEAC, and hemogram data in this study. The AOPP was significantly different at 24 h after laparoscopy; it could be related to the pneumoperitoneum, which causes a decrease in splanchnic blood flow through both systemic and local effects in a pressure- and time-dependent manner [28]. There is evidence in animal studies that pneumoperitoneum causes end-organ ischemia and damage. Abdominal insufflation and increased intra-abdominal pressure can cause significant organ ischemia followed by reperfusion injury upon gastric emptying, resulting in an imbalance between oxidants and antioxidants [28]. Also, it has been shown in canines that heating CO_2_ can generate a greater inflammatory response and formation of reactive oxygen species at the plasma and peritoneal levels [29]. In the same way, the thiol concentrations did not contribute to differentiating the groups, supporting the assumption that minimizing operative time and iatrogenic lesions are necessary to reduce the oxidative protein damage [10,28].

Considering the oxidant and antioxidant biomarkers used in this study, it could be assumed that no oxidative stress was present in the patients that underwent the different OVH techniques. Similarly, no changes in total serum oxidation state and total antioxidant capacity were observed in the previous study [30]. A similar protocol has been used to evaluate pyometra in queens, and it was considered efficient [31].

In general, the duration of the surgical stress response is proportional to the injury and complications [25]. As observed in this study, compared to the celiotomy, the video-assisted procedure improves the cosmetic effect, reduces the risk of complications due to a smaller incision, which significantly reduces recovery time [5,6], and promotes a less intense response to surgical stress [4,5], as related before.

It should be noted that cortisol concentrations were not measured in this study. Further research should incorporate cortisol levels alongside the analytes assessed here to provide a more comprehensive understanding on the utility of these markers in characterizing surgical stress.

## 5. Conclusions

The OVH techniques produced a similar inflammatory and oxidative stress response, but the video-assisted technique promoted earlier clinical recovery. The bipolar device has been presented as a safe device, less injurious than the traditional method of suspensory ligament rupture and ligature.

## Figures and Tables

**Table 1:** Surgery time, incision size, and time to first solid intake, urination, and defecation after canine OVH via ventral median celiotomy and ligature (CelioSut), ventral median celiotomy and bipolar coagulation (CelioBip), and video-assisted technique with two ports and bipolar coagulation (VidBip).

	CelioSut	CelioBip	VidBip	*p* Value
	M ± SD	M ± SD	M ± SD	
Surgery time (min) ^a^	21.44 ± 5.47 A	20.20± 5.02 A	58.40 ± 16.17 B	*p* < 0.01
Incision size (cm) ^a^	5.96 ± 0.66 A	5.95 ± 1.26 A	2.91 ± 0.62 B	*p* < 0.01
	**Md (Interquartile Range)**	**Md (Interquartile Range)**	**Md (Interquartile Range)**	
First solid intake (h) ^b^	11.82 (5.91–13.38) A	6.55 (6.23–10.38) A	1.88 (1.21–3.77) B	*p* < 0.01
First urination (h) ^b^	7.38 (5.72–8.97) A	6.59 (3.5–7.06) AB	4.00 (2.83–5.46) B	*p* = 0.03
First defecation (h) ^b^	18.77 (11.95–25.55) A	9.19 (6.8–22.15) A	11.08 (10.19–12.25) A	*p* = 0.28

The normality of data distribution was verified with the Shapiro–Wilk test. ^a^ Parametric data (means [M] ± standard deviation [SD]). ^b^ No parametric data (median [Md] and interquartile range [Q1–Q3]). Capital letters indicate statistical differences between groups.

**Table 2:** Rectal temperature, respiratory rate, and heart rate after canine OVH via ventral median celiotomy and ligature (CelioLig), ventral median celiotomy and bipolar coagulation (CelioBip), and video-assisted technique with two ports and bipolar coagulation (VidBip).

Times	CelioLig	CelioBip	VideoBip	*p* Value **
Rectal temperature (°C)				
Basal	38.40 a	38.55 a	38.75 a	0.29
2	37.40 b	37.70 b	38.05 b	0.06
6	38.00 bAB*	37.90 bB	38.20 abA	0.04
12	38.20 aAB	38.05 bB	38.65 aA	0.04
24	38.20 a	38.15 ab	38.65 a	0.10
48	38.50 a	38.25 ab	38.65 a	0.06
72	38.45 a	38.30 a	38.50 a	0.30
*p* value **	*p* < 0.01	*p* = 0.03	*p* = 0.01	
Respiratory rate (mpm)				
Basal	38	34	34	0.42
2	28	28	24	0.79
6	31	32	28	0.33
12	36	34	29	0.07
24	36 A*	38 A	28 B	0.01
48	34	32	28	0.26
72	32	34	32	0.48
*p* value **	0.06	0.33	0.06	
Heart rate (bpm)				
Basal	116	108	120 a*	0.60
2	110	100	110 a	0.46
6	118 A**	122 A	96 bB	0.01
12	128 A	114 A	98 bB	0.01
24	124 A	124 A	96 bB	0.01
48	112 A	112 A	102 abB	0.03
72	116	104	102 ab	0.18
*p* value **	0.27	0.38	0.01	

The normality of data distribution was verified with the Shapiro–Wilk test. Later, the data were investigated via the * Dunn–Bonferroni and ** Kruskal–Wallis Tests. Capital letters and small letters indicate statistical difference between groups.

## Data Availability

The raw data supporting the conclusions of this article will be made available by the authors on request.
